# Supplementary material for: Mutations in PCYT2 disrupt etherlipid biosynthesis and cause a complex hereditary spastic paraplegia
Source: Brain. 2019 Oct 22;142(11):3382–97. doi: 10.1093/brain/awz291 (PMC6821184; doi:10.1093/brain/awz291)
Supplement: awz291_Supplementary_Data [file awz291_supplementary_data.zip › awz291-Suppl_data/Supplementary_Data4.zip!/Supplementary information 4 - plasma lipidomics.zip!/Plasma lipidomics/Control vs Patient2/Statistical plots and data/LPC species/Boxplots all.pdf]

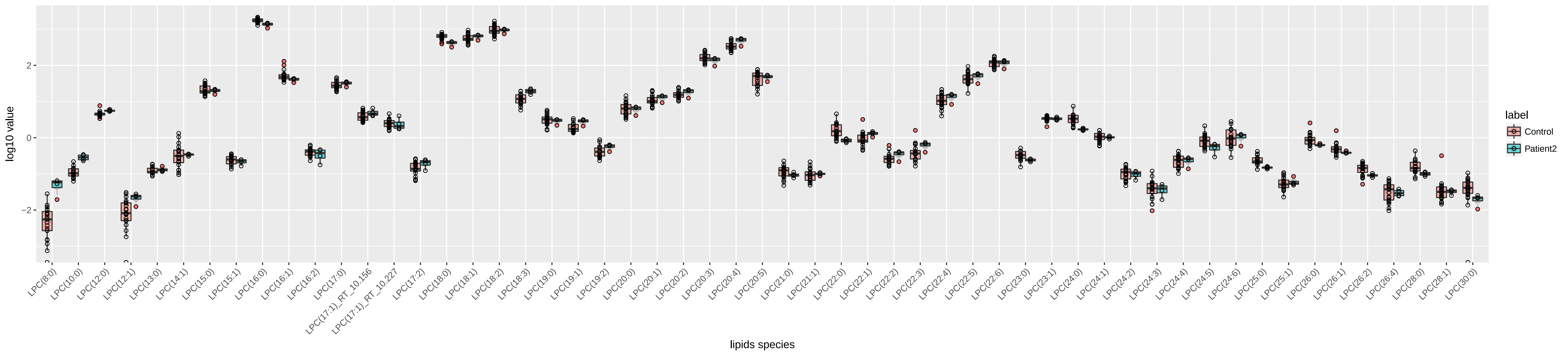

log10 value
2
0
-2
lipids species
label
Control
Patient2
LPC(8:0)
LPC(10:0)
LPC(12:0)
LPC(12:1)
LPC(13:0)
LPC(14:1)
LPC(15:0)
LPC(15:1)
LPC(16:0)
LPC(16:1)
LPC(16:2)
LPC(17:0)
LPC(17:1)_RT_10.156
LPC(17:1)_RT_10.227
LPC(17:2)
LPC(18:0)
LPC(18:1)
LPC(18:2)
LPC(18:3)
LPC(19:0)
LPC(19:1)
LPC(19:2)
LPC(20:0)
LPC(20:1)
LPC(20:2)
LPC(20:3)
LPC(20:4)
LPC(20:5)
LPC(21:0)
LPC(21:1)
LPC(22:0)
LPC(22:1)
LPC(22:2)
LPC(22:3)
LPC(22:4)
LPC(22:5)
LPC(22:6)
LPC(23:0)
LPC(23:1)
LPC(24:0)
LPC(24:1)
LPC(24:2)
LPC(24:3)
LPC(24:4)
LPC(24:5)
LPC(24:6)
LPC(25:0)
LPC(25:1)
LPC(26:0)
LPC(26:1)
LPC(26:2)
LPC(26:4)
LPC(28:0)
LPC(28:1)
LPC(30:0)
